# Symmetric Supercapacitor Electrodes from KOH Activation of Pristine, Carbonized, and Hydrothermally Treated *Melia azedarach* Stones

**DOI:** 10.3390/ma10070747

**Published:** 2017-07-04

**Authors:** Carlos Moreno-Castilla, Helena García-Rosero, Francisco Carrasco-Marín

**Affiliations:** Departamento de Química Inorgánica, Universidad de Granada, 18071 Granada, Spain; helegaro@gmail.com (H.G.-R.); fmarin@ugr.es (F.C.-M.)

**Keywords:** *Melia azedarach* stones, biomass wastes, activated carbons, supercapacitor electrodes, energy storage

## Abstract

Waste biomass-derived activated carbons (ACs) are promising materials for supercapacitor electrodes due to their abundance and low cost. In this study, we investigated the potential use of *Melia azedarach* (MA) stones to prepare ACs for supercapacitors. The ash content was considerably lower in MA stones (0.7% ash) than that found in other lignocellulosic wastes. ACs were prepared by KOH activation of pristine, carbonized, and hydrothermally-treated MA stones. The morphology, composition, surface area, porosity, and surface chemistry of the ACs were determined. Electrochemical measurements were carried out in three- and two-electrode cells, 3EC and 2EC, respectively, using 1 M H_2_SO_4_ as the electrolyte. The highest capacitance from galvanostatic charge-discharge (GCD) in 2EC ranged between 232 and 240 F·g^−1^ at 1 A·g^−1^. The maximum energy density reached was 27.4 Wh·kg^−1^ at a power density of 110 W·kg^−1^. Electrochemical impedance spectroscopy (EIS) revealed an increase in equivalent series resistance (ESR) and charge transfer resistance (R_CT_) with greater ash content. Electrochemical performance of MA stone-derived ACs was compared with that of other ACs described in the recent literature that were prepared from different biomass wastes and results showed that they are among the best ACs for supercapacitor applications.

## 1. Introduction

Supercapacitors or electrochemical capacitors are energy storage devices with ideal characteristics for the rapid storage and release of energy and for a long-term cycling life [1,2,3]. This capability is mainly achieved through a non-faradic mechanism by the separation of ions across a very small distance in the electrochemical double-layer (EDL) at the electrolyte/electrode interface, and it is dependent on the surface area of the electrode and the accessibility of the electrolyte to its porosity. EDL capacitance can be improved by reversible faradic reactions that introduce pseudocapacitance [1,2,3].

Porous carbon materials are widely used as supercapacitor electrodes due to their high surface area, well-developed porosity, high conductivity, good physicochemical stability, and the presence of functionalities, such as oxygen and nitrogen complexes, which can introduce pseudocapacitance phenomena [2,3,4,5]. ACs are a highly interesting type of porous carbon material for use as supercapacitor electrodes because they can be obtained from readily available, inexpensive, and renewable raw materials, including biomass residue and waste. Their utilization is also a form of confronting the problem of waste disposal.

Thus, various studies have recently been published on the preparation of ACs from these raw materials for application as supercapacitor electrodes [5,6,7,8,9,10,11,12,13,14,15,16,17,18,19,20,21,22,23,24]. The biomass wastes employed have mostly been lignocellulosic, although other types of material, such as algae (composed of proteins and carbohydrates), have been used. Table 1 summarizes the electrochemical characteristics of these ACs, which were all prepared by chemical activation, mainly by KOH [25].

*Melia azedarach*, also known as Chinaberry, is a widely planted urban ornamental tree in many Mediterranean countries because of the large shadow it casts in the summer and its carbon sink potential [26]. We investigated the usefulness of MA stones to obtain ACs for application as supercapacitor electrodes, preparing three AC series by KOH activation of pristine, carbonized, and hydrothermally treated MA stones, designated as MA, CMA, and HMA, respectively. The ACs obtained were characterized to determine their morphology, composition, surface area, porosity, surface chemistry, and their electrochemical characteristics (in 3EC and 2EC with 1 M H_2_SO_4_ as the electrolyte).

## 2. Results and Discussion

**Figure 1:**
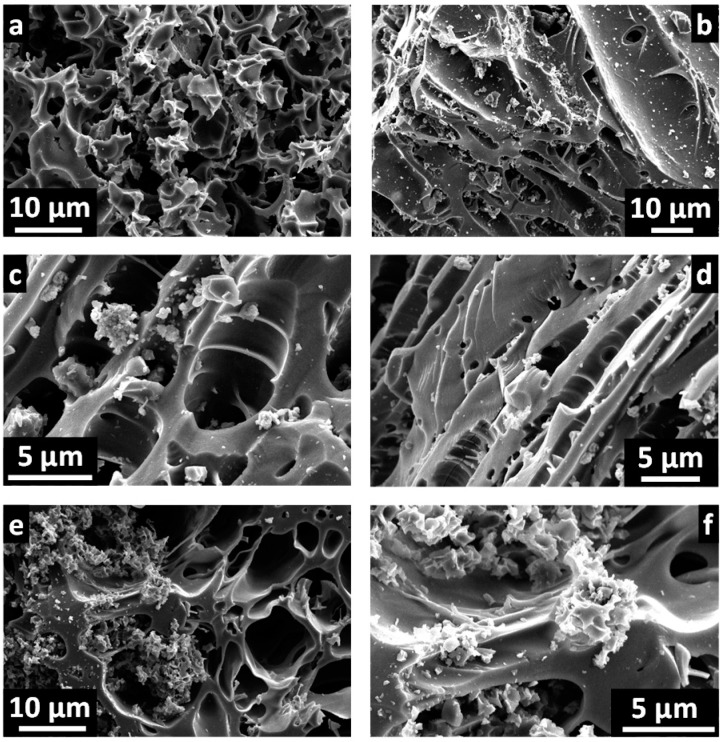
ESEM images of activated carbons: (**a**) MA2; (**b**) CMA2; (**c**) CMA4; (**d**) HMA100-2; (**e**) HMA150-2; and (**f**) HMA200-2. ESEM: environmental scanning electron microscopy; MA: *Melia azedarach* stones; CMA: carbonized *Melia azedarach* stones; HMA: hydrothermally treated *Melia azedarach* stones.

Environmental scanning electron microscopy (ESEM) images in Figure 1 and Figure S1 (in Supplementary materials) reveal the morphology of ACs and precursors, respectively. The carbonized *Melia azedarach* stone (CMA) precursor showed very large pores and channels (Figure S1a,b), generated by the evolution of the volatile matter during the carbonization of MA stones. Figure S1c–f depicts the precursors obtained by hydrothermal treatment of MA at different temperatures. Sample HMA200 was the only one that contained numerous sphere-like microparticles on the surface of some larger particles, generated during the hydrothermal treatment at 200 °C of the saccharides contained in MA stones [5,27].

Morphologically, the ACs were composed of irregular, more or less divided particles, which contained large pores and conchoidal cavities caused by brittle fracture of the precursor particles during KOH activation.

The ash content and ultimate analysis of samples are displayed in Table 2. None of the samples contained sulfur. Notably, the ash content of MA stones was very low, considerably below that of other lignocellulosic waste biomasses used to prepare ACs [8,28]. This is important for the selection of potential wastes to prepare activated carbons [29]. The carbonization of MA to obtain CMA increased the ash and C content and reduced the other elements due to volatile matter removal.

The hydrothermal treatment of MA to obtain HMA produced a slight reduction in ash content and increase in C content with the rise in treatment temperature from 100 to 200 °C due to the loss in oxygen content [27]. The ash content was lower in all ACs from the MA and CMA series than in their corresponding precursors because the activation included a final hydrochloric acid treatment to remove the potassium compounds formed. The ash content also decreased with higher KOH/precursor mass ratio or hydrothermal treatment temperature. The ash content was higher in ACs from the HMA series than in their respective precursors, despite the final HCl treatment, indicating that the inorganic compounds of the precursors were less accessible or reactive to this acid treatment after KOH activation. The influence of ash accessibility on its removal is indicated by the finding of a reduction in its content with the higher hydrothermal treatment temperature of the precursor, because this increased the surface area of the ACs (vide infra). The lowest ash content was observed in ACs from the MA precursor.

N and O measured by ultimate analysis and XPS can be considered as the total and surface contents, respectively, giving an idea of their distribution in the ACs. With regard to the N content, N_XPS_ was higher than the total N content in the more activated samples (MA2, MA3, and CMA4) and in those receiving hydrothermal treatment. A similar trend was observed for the O content in CMA4, HMA150-2, and HMA200-2 samples.

Figure S2 depicts the N_2_ adsorption isotherms on the ACs, which were of Type I [30], typical of microporous solids; however, there was a slight increase in N_2_ uptake with greater relative pressure after micropore filling, indicating the presence of mesopores below 4 nm in size [31]. N_2_ and CO_2_ adsorption isotherm results are compiled in Table 3. The surface area, W_0_(N_2_), and V_0.95_ increased with the higher KOH/precursor mass ratio or hydrothermal treatment temperature of MA, while the CMA4 sample showed the highest S_BET_ value (ca. 2000 m^2^·g^−1^). CO_2_ adsorption at 0 °C yielded the volume of narrow micropores (below approximately 0.7 nm in width), while N_2_ adsorption at −196 °C gave the total micropore volume if there were no micropores that were very narrow or had constricted entrances [32,33]. Table 3 shows that W_0_(N_2_) < W_0_(CO_2_) for ACs obtained with lower KOH/precursor mass ratios, attributable to the presence in these samples of very narrow micropores or micropore constrictions. However, they were widened or the constrictions disappeared at KOH/precursor mass ratios >2. W_0_(N_2_) > W_0_(CO_2_) for all ACs in the HMA series. ACs in the MA and CMA series were more microporous (between 80–87%) in comparison to ACs in the HMA series (73%).

**Figure 2:**
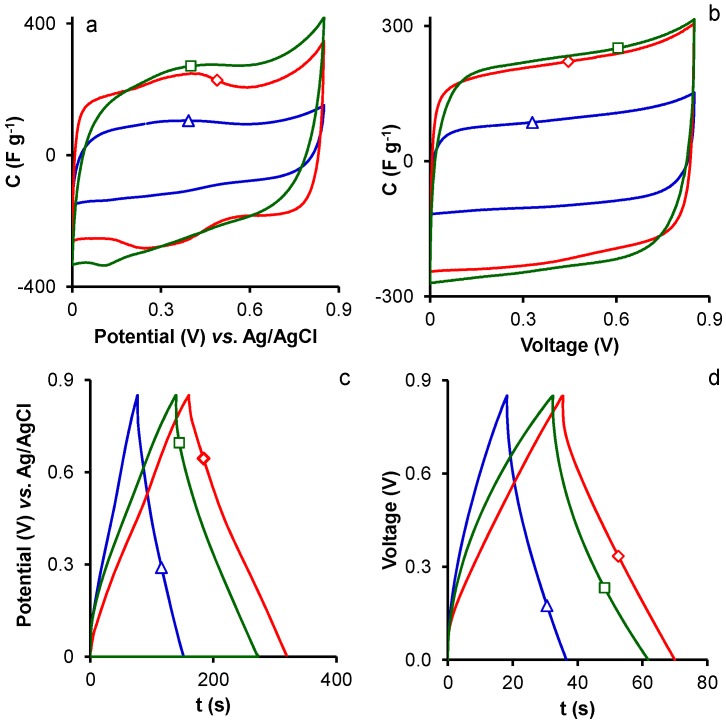
CVs at 2.5 mV·s^−1^ of samples: (**a**,**b**) MA1 (△), MA2 (□), and MA3 (◇); and (**c**,**d**) GCDs at 1 A·g^−1^ of samples MA1 (△), MA2 (□), and MA3 (◇).

Cyclic voltammetries (CVs) at 2.5 mV·s^−1^ obtained in 3EC and 2EC are depicted in Figure 2 and Figure S3. CVs obtained with 3EC are very useful for analyzing faradic reactions and voltages at a single surface [34,35]. Thus, they had a quasi-rectangular shape, with a clear faradic hump at a cell potential below around 0.4 V, indicating the presence of pseudocapacitance phenomena attributed to the oxygen and nitrogen functionalities [5] contained by all ACs under study. However, CVs in 2EC showed rectangular shapes, with no pseudocapacitance effects.

CVs in 2EC and 3EC showed that voltammograms retained a rectangular-like shape and their capacitance decreased with an increase in the scan rate (Figures S4–S6), attributable to the inaccessibility of the internal surface of narrower micropores at high scan rates due to ion diffusion [36]. The pseudocapacitance effects observed in 3EC also disappeared at higher scan rates.

GCDs in 2EC and 3EC at 1 A·g^−1^ (Figure 2 and Figure S3) showed a triangular shape, and their gravimetric and areal capacitances are exhibited in Table 4. Values were always lower in 2EC than in 3EC, as previously reported at low current densities [34]. The gravimetric capacitance increased with higher KOH/precursor mass ratio in the MA and CMA series due to the resulting increase in surface area and porosity. In samples from the HMA series, C increased with a rise in the hydrothermal treatment temperature from 100 to 150 °C, but was not affected by a further rise up to 200 °C.

MA2, MA3, and CMA4 samples showed similar C values in 2EC, in the 232–240 F·g^−1^ range. This was the higher capacitance obtained in the present study and was within the range reported for the top six ACs (229–380 F·g^−1^ at 1 A·g^−1^) compiled in Table 1. The coulombic efficiency in all cases was 100% at 1 A·g^−1^ and it decreased up to ~97% at the higher current density due to the cell resistance. Although the gravimetric capacitance of these three samples was similar, their areal capacitance, C_A_, was very different, attributable to the pseudocapacitance effects of their N and O contents. Thus, the C_A_ value was considerably higher for MA2 (22.5 μF·cm^−2^) than for CMA4 (12.2 μF·cm^−2^), which was the AC with the lowest N and O contents of these three samples. Gravimetric capacitance decreased when higher current density increased (Figure S7), and the retention capacitance at 11.1 A·g^−1^ was 26%, 21%, and 32%, for MA2, MA3, and CMA4 samples, respectively.

**Figure 3:**
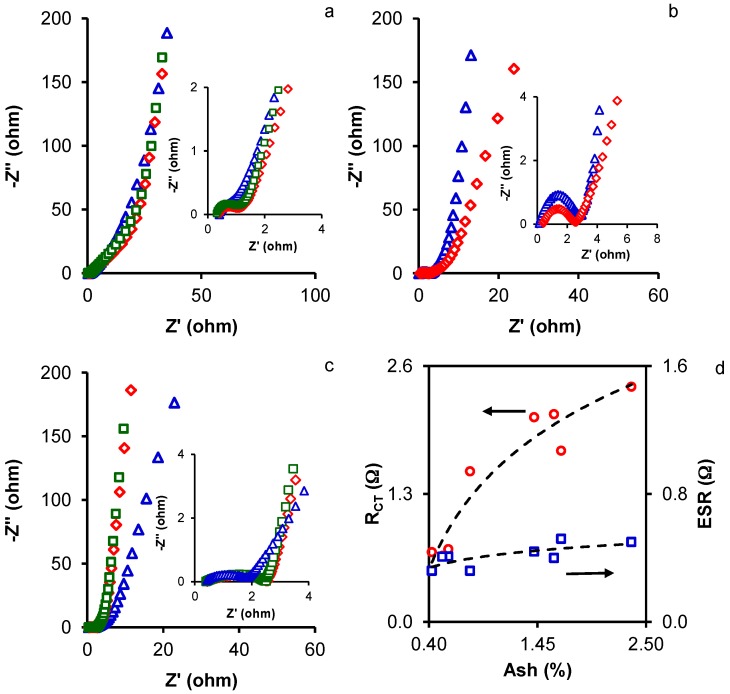
Nyquist plots of samples (**a**) MA1 (△), MA2 (◇), and MA3 (□); (**b**) CMA2 (△), and CMA4 (◇); and (**c**) HMA100-2 (△), HMA150-2 (◇), and HMA200-2 (□). (**d**) The relationship between ESR (□) and R_CT_ (○) with the ash content of the activated carbons.

EIS measurements were performed to investigate the charge kinetic properties of the ACs towards the capacitive behavior. Figure 3 depicts the Nyquist plots, showing that all samples exhibited typical characteristics of porous carbon electrodes [15,21,22,37]. In the high-frequency region, the first intersection point on the real axis (Z’) was the equivalent series resistance, ESR, which was related to three resistances: the intrinsic resistance of the active material, the electrolyte resistance, and the active material/current collector interface. Results, shown in Table 4, can be considered to approximately reflect the conductive properties of the activated carbon electrodes for comparison purposes, because the same electrolyte, collectors, and technique were used to assemble the cell [38]. ESR values were very low, ranging between ~0.3 and 0.5 Ω, and they tended to decrease with higher KOH/precursor mass ratio or hydrothermal temperature. Thus, the lowest ESR values in the AC series were 0.32 Ω (MA3 and HMA200-2) and 0.40 (CMA4).

In the high-medium frequency region, a semicircle loop was observed in association with charge transfer processes, indicating the presence of pseudocapacitance phenomena, which were also detected in the CVs in 3EC (vide supra) due to the N and O functionalities on the AC surfaces [5,23]. The interfacial charge transfer resistance, R_CT_, caused by EDL capacitance and faradic reactions was estimated from the two intercepts of the semicircle with the real axis (semicircle diameter) [15], and the values obtained are displayed in Table 4. R_CT_ values were lower in ACs from the MA series than from the other two series. The lowest R_CT_ values were found in the most highly activated AC in each series (MA3, CMA4, and HMA200-2) due to their increased surface area and micropore volume, which enhanced the accessibility of ions to the electrode porosity and the charge transfer.

Notably, both ESR and R_CT_ tended to decrease with lower ash content of the ACs (Figure 3d). This indicated that the ash removal facilitated the electron mobility through the carbon electrodes decreasing the interparticle contact resistance and then the ESR value. On the other hand, the ash removal increased the accessibility of the electrolyte to the microporosity facilitating the EDL formation and making to decrease the R_CT_ value.

In the low-frequency region, the imaginary part of the impedance (Z") sharply increased and the plots tended to a vertical line, which is characteristic of capacitive behavior. The maximum capacitance (C_max_) of the ACs was obtained at the lowest frequency (1 mHz) using Equation (2). Results in Table 4 showed that C_max_ was similar or close to the C values yielded by GCD measurements in 2EC. Variations in the imaginary part of the capacitance (C") against the frequency (Figure S8) define the transition frequency between a pure capacitive and a pure resistive behavior. The relaxation time constant (τ) can be obtained from the frequency, f_0_, at the maximum of these curves by the equation τ = 1/2πf_0_ [24]. The relaxation time constant is a quantitative measure of the speed with which the device can be discharged [35]. Results in Table 4 show that τ generally decreased with higher activation in each AC series 

**Figure 4:**
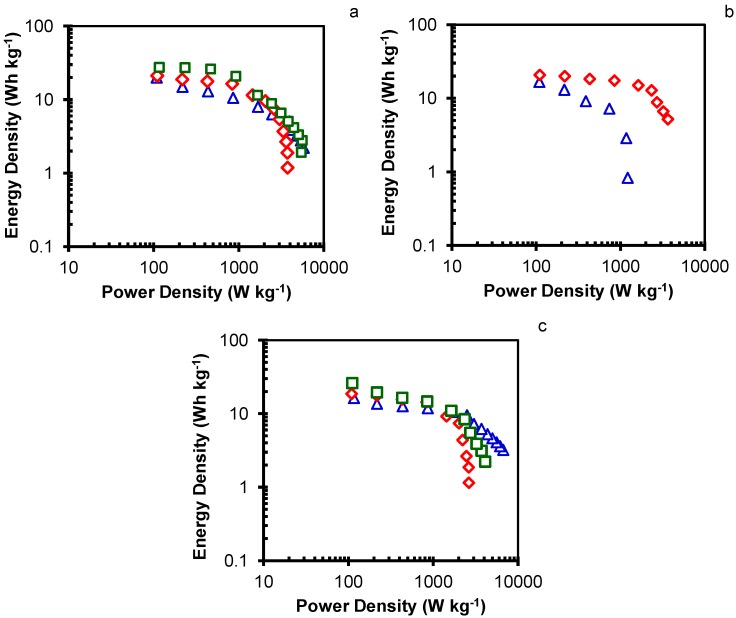
Ragone plots of samples (**a**) MA1 (△), MA2 (◇), and MA3 (□); (**b**) CMA2 (△), and CMA4 (◇); and (**c**) HMA100-2 (△), HMA150-2 (◇), and HMA200-2 (□).

The Ragone plots of the ACs are displayed in Figure 4 and reveal the dependence between energy and power densities. The maximum energy density was released at the lowest power density (110 W·kg^−1^), as shown in Table 5. The maximum energy density increased with higher activation in each AC series, attributable to the increase in surface area and micropore volume produced.

The maximum value reached was 27.4 Wh·kg^−1^ for sample MA3. Notably, the energy density of the symmetrical capacitor based on MA3 was better than that reported for most of the commercially-available supercapacitors (less than 10 Wh·kg^−1^) [39] and most of the recently-reported ACs derived from biomass wastes (Table 1). The energy released decreased at higher power density (Figure 4); thus, the energy densities of MA3, CMA4, and HMA200-2 samples were decreased to 2.8, 3.6, and 2.2 Wh·kg^−1^ at power densities of ~5.6, 3.6, and 4.1 kW·kg^−1^, respectively.

The long-term stability of AC-based symmetric supercapacitors is considered a key factor for their practical application. This stability was evaluated by the voltage-holding or floating test, which was recently established [22,40,41,42] as a reliable alternative to the traditional method of GCD cycles at constant current density over several thousands of cycles. The floating test was carried out by applying a critical voltage cell (0.85 V), and by occasionally cycling the cell between 0.85 and 0 V at a constant current density of 1 A·g^−1^, determining the respective capacitance and the resistance from the IR voltage drop. During the voltage-holding procedure, three cycles were applied every 10 h and this sequence was repeated 20 times [42], giving a total floating time of 200 h.

**Figure 5:**
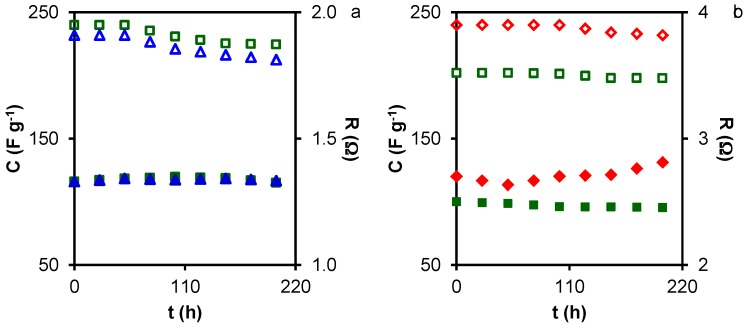
Floating tests of samples: (**a**) MA2 (△), and MA3 (□); (**b**) CMA4 (◇), and HCM200-2 (□). Gravimetric capacitance (open symbols) and resistance (closed symbols).

Figure 5 depicts the results of the floating test for samples from the three series that showed the higher capacitance and energy release (MA2, MA3, CMA4, and HMA200-2). The capacitance of MA2 and MA3 (Figure 5a) did not change during ~50 h of voltage-holding and subsequently started to decrease, showing a capacitance decay of 8% (MA2) and 6% (MA3) after 200 h of floating time. The resistance was practically constant throughout this time. In CMA4 and HMA200-2 samples (Figure 5b), the capacitance was constant up to ~100 h of voltage-holding, showing a capacitance decay of 1% and 2%, respectively, after 200 h. In CMA4, the resistance slightly increased after 100 h, indicating a slight degradation of the electrodes. The results for these ACs indicated their good stability as potential supercapacitor electrodes, especially in the case of CMA4.

## 3. Materials and Methods

### 3.1. Preparation of Activated Carbons

MA fruits were collected from trees on the campus of Granada University. After removal of the peel and pulp, the stones were washed with distilled water and dried at 110 °C. MA stones were carbonized and hydrothermally treated to obtain CMA and HMA samples, respectively. Carbonization of MA stones was carried out at 800 °C for 1 h in a N_2_ flow. HMA samples were prepared by heating a mixture of 50 g MA stones and 100 mL distilled water in a 250 mL Teflon-lined autoclave at 100–150 °C for 24 h. These samples are designated as HMAt, with t being the treatment temperature. Subsequently, MA, CMA, and HMAt samples were KOH activated. For this purpose, they were drop-wise impregnated with a concentrated KOH solution under an infrared lamp (ca. 70 °C) to yield a KOH/raw material mass ratio between 1 and 4. The impregnated raw material was then dried and carbonized at 800 °C for 1 h in a N_2_ flow, followed by treatment with 1 M HCl and washing with distilled water until the absence of chloride ions in the washing water. These ACs are designated as MAx, CMAxm and HMAt-x, respectively, with x indicating the KOH/precursor mass ratio. In all cases, KOH-activation of the precursors produced their breakdown, and the powdered ACs obtained were sieved to a size between 0.15 and 0.25 mm for utilization in all measurements.

### 3.2. Characterization

The morphology of samples was examined by environmental scanning electron microscopy (ESEM) using a Quanta 400 equipment (FEI, Thermo Fiscer Sci., Hillsboro, OR, USA). Ultimate analysis (C, H, N, and S) was carried out using a Thermo Finnigan elemental analyzer (FlashEA1112, Thermo Fischer Sci., Delft, The Netherland). Ash content was determined by heating the sample at 800 °C in air until constant weight using a thermobalance (TGA-50H, Shimadzu Co., Kyoto, Japan). N and O surface contents were determined by X-ray photoelectron spectroscopy (XPS) using a MgK_α_ X-ray source, hγ = 1253.6 eV, (ESCA5701, Physical Electronics Inc., Chanhassen, MN, USA). Each spectral region of the photoelectron interest was scanned several times to obtain good signal-to-noise ratios. The C_1s_ peak at a binding energy (BE) of 284.6 eV was used as the internal standard to obtain the number of components, position of peaks, and peak areas.

N_2_ and CO_2_ adsorption isotherms were measured at −196 and 0 °C, respectively, using a gas sorption equipment (Autosorb 1, Quantachrome, Boynton Beach, FL, USA) after outgassing samples overnight at 10^−6^ mbar and 110 °C. The Brunauer-Emmett-Teller (BET) equation was applied to the N_2_ adsorption isotherm to obtain the surface area, S_BET_, and the DR equation was applied to both isotherms to obtain the micropore volume, W_0_, and micropore width, L_0_. The total pore volume, V_0.95_, was obtained from the amount of liquid N_2_ adsorbed at p/p_0_ = 0.95 and the percentage of micropores from [W_0_(N_2_)/V_0.95_] × 100.

### 3.3. Electrochemical Measurements

Electrochemical measurements were investigated at room temperature with 1 M H_2_SO_4_ as the electrolyte in both 2EC (Teflon-Swagelock-type) and 3EC. Working electrodes were prepared from a well-mixed slurry of the ACs (90 wt %) with polytetrafluoroethylene (PTFE) emulsion (10 wt %) as a binder. This slurry was pressed at 3 bar on graphite paper disks (2EC) or graphite foil (3EC) and dried overnight in an oven at 120 °C. The discs had an area of ca. 0.50 cm^2^ (φ 8 mm) and contained the same amount of active material, ca. 4 mg, whereas the graphite foil contained ca. 20 mg of active material. Electrodes were immersed in the electrolyte for five days before their assembly in the cells. The 2EC was a symmetric device comprising two equal electrodes with the same mass of active material separated by a porous fibrous separator impregnated with the electrolyte solution. The 3ECs comprised the above slurry pasted on graphite paper as working electrode, with reference electrode (Ag/AgCl), and counter electrode (Pt wire).

Cyclic voltammetry (CV), GCD, and EIS measurements were done using a potentiostat (VMP-300, BioLogic, Seyssinet-Pariset, France). CVs and GCDs were performed in 2EC and 3EC in the 0–0.85 V range, at scan rates between 0.5 and 30 mV·s^−1^ for CVs and current densities between 0.14 and 11.1 A·g^−1^ for GCDs. EIS was carried out in 2EC within the frequency range of 1 mHz to 100 kHz with a sinusoidal signal amplitude of 10 mV.

Gravimetric capacitances, *C* (F/g), were obtained from the discharge curves of the GCDs by Equation (1):

(1) $$C=\;I_{d}\Delta t/m\Delta V$$

where $I_{d}$ is the discharge current, Δ*t* the discharge time, m the total mass of the AC in the electrodes, and ΔV the voltage interval after the ohmic drop. The coulombic efficiency (%) was calculated from the discharge and charge time, *t_d_* and *t_c_*, respectively, by the formula (*t_d_/t_c_*) × 100. The capacitance value from EIS, *C*, was obtained by Equation (2):

(2) $$C=\;-Z\prime \prime /2\pi f\;|Z{|}^{2}$$

where *f* is the frequency and $|Z{|}^{2}=\;{Z\prime }^{2}+\;Z{\prime \prime }^{2}$, with Z′ and *Z*″ being the real and imaginary parts of the complex impedance, respectively.

For performance comparisons, the gravimetric capacitances in 2EC obtained from the above equations were multiplied by four to obtain the expression per single electrode, which is the 3EC equivalent [43]. However, per convention, the gravimetric capacitances obtained from Equation (1) in 2EC, C_2EC_, were used to calculate the energy density in the Ragone plot using the equation $E=\;C_{2EC}{\left(\Delta V_{d}\right)}^{2}/2$, where $\Delta V_{d}$ is the operation voltage taken as $\Delta V_{d}=\;V_{max}-\;IR_{drop}$. Power density was calculated from P= E/Δt [44,45].

## 4. Conclusions

In this study, three series of ACs were prepared by KOH activation of *Melia azedarach* (MA) stones (MA series), carbonized MA stones (CMA series) and hydrothermally-treated MA stones (HMA series). MA stones had very low ash content, 0.7%, which is of great importance for AC preparation. ACs from MA series showed the lower ash content (0.43–0.59%). ACs from the three series contained different amounts of N and O functionalities. The highest surface area obtained was ca. 2000 m^2^·g^−1^ (CMA4). ACs from HMA series had a lower percentage of microporosity than those from the other two series. The higher gravimetric capacitance was in the 232–240 F·g^−1^ range at 1 A·g^−1^, and sample MA2 showed the highest areal capacitance 22.5 μF·cm^−2^. CVs in 3EC and EIS in 2EC showed pseudocapacitance phenomena attributed to the N and O functionalities. Notably, the ESR and R_CT_ tended to decrease when the ash content of the AC electrode decreased, which indicated the importance of controlling the mineral content of the raw material used to prepare ACs for supercapacitor applications. The maximum energy density reached (MA3) was 27.4 Wh·kg^−1^ at a power density of 110 W·kg^−1^. A cycling test based on floating was used to investigate the long-term stability and results showed that devices could be charged and discharged without notable degradation. Electrochemical performance of MA stone-derived ACs was compared with that of other ACs described in the recent literature that were prepared from different biomass wastes, mainly of lignocellulosic and algae origins, and results showed that they are among the best ACs for supercapacitor applications.

## Figures and Tables

**Table 1:** Electrochemical capacitance performances of activated carbons from biomass wastes.

Biomass Precursor	Activation	S_BET_ (m^2^·g^−1^) ^a^	Capacitances at 1 A·g^−1^ (F·g^−1^ or μF·cm^−2^)	Energy Density (Wh·Kg^−1^) at Power Density (kW·kg^−1^)	Electrolyte	Reference
Lotus seed pods	KOH	1813	380 or 20.9	12.5 at 0.26	6 M KOH	[9]
*Eulaliopsis binata*	KOH	2273	360 or 15.8	nd ^b^	6 M KOH	[10]
Cabbage leaves	KOH	3012	336 or 11.2	nd	2 M KOH	[11]
*Euonimus japonica* leaves	KOH	1268	275 or 21.7	5.0 at 8.6	6 M KOH	[7]
Willow catkins	KOH	1115	275 or 24.7	8.8 at 0.05	6 M KOH	[12,23]
Dragonfruit skin	KOH	911	240 or 26.3	nd	2 M KOH	[13]
Rapeseed	ZnCl_2_	682	229 or 33.5	13.5 at 0.4 ^c^	6 M KOH	[14]
Peanut shells	KOH	1277	228 or 17.9	6 at 0.8	6 M KOH	[8]
Fir sawdust	KOH	2395	225 or 9.4	8.4 at 0.25	6 M KOH	[24]
Soybean residue	KOH	1340	220 or 16.4	12 at 0.025	1 M H_2_SO_4_	[5]
*Enteromorpha prolifera*	700 °C (N_2_ atm.)	422	216 or 51.2	nd	6 M KOH	[15]
Chinese fir sawdust	KOH	2294	213 or 9.3	7.8 at 0.25	6 M KOH	[16]
*Momordica grosvenori* skin	KOH	597	208 or 34.8	nd	2 M KOH	[13]
*Firmiana* catkins	KOH	287	195 or 67.9	nd	2 M KOH	[13]
Onion husks	K_2_CO_3_	2571	188 or 7.3	47.6 at 0.67	1 M TEABF_4_/AN	[17]
Willow leaves	ZnCl_2_	1031	172 or 16.7	nd	6 M KOH	[18]
Hemp stem	KOH	2801	167 or 6.0	19.8 at 21	1.8 M TEMABF_4_/PC	[19]
Bamboo shells	Na_2_CO_3_-K_2_CO_3_	843	164 or 19.5	nd	1 M H_2_SO_4_	[20]
Rice husk	KOH	2696	120 or 4.5	5.1 at 0.05	6 M KOH	[21]
Pinecones	KOH	1515	78 or 5.1	2.7 at 0.5	1 M Na_2_SO_4_	[22]

^a^ S_BET_: BET surface area; ^b^ nd: non-determined; ^c^ in 0.5 M Na_2_SO_4_.

**Table 2:** Ash content (%), ultimate analysis (wt %) in dry basis, and N and O contents (wt %) from X-ray photoelectron spectroscopy (XPS) of the raw material, precursors, and activated carbons.

Sample	Ash	C	H	N	O ^a^	N_XPS_	O_XPS_
MA	0.70	50.60	6.90	1.28	40.52	nd	nd
MA1	0.59	88.17	0.26	0.73	10.25	0.38	7.60
MA2	0.53	86.62	0.27	0.76	11.82	1.03	10.80
MA3	0.43	85.62	0.34	0.86	12.75	1.07	11.72
CMA	5.28	85.81	0.79	1.32	6.80	1.06	6.51
CMA2	2.36	85.37	0.28	0.44	11.55	0.32	8.43
CMA4	1.61	90.07	0.24	0.53	7.55	0.65	8.90
HMA100	0.62	53.58	7.46	1.38	36.96	nd	nd
HMA150	0.63	61.33	7.68	1.42	28.94	nd	nd
HMA200	0.63	68.17	7.94	1.73	21.53	nd	Nd
HMA100-2	1.68	85.75	0.32	0.65	11.60	0.71	7.81
HMA150-2	1.42	88.26	0.20	0.70	9.42	0.85	10.44
HMA200-2	0.80	89.28	0.20	0.79	8.93	0.89	10.95

^a^ by difference.

**Table 3:** Surface area and pore texture of the activated carbons.

Sample	S_BET_ (m^2^·g^−1^)	W_0_(N_2_) (cm^3^·g^−1^)	W_0_(CO_2_) (cm^3^·g^−1^)	L_0_(N_2_) (nm)	L_0_(CO_2_) (nm)	V_0.95_ (cm^3^·g^−1^)	Micro ^a^ (%)
MA1	829	0.32	0.41	0.80	0.63	0.40	80
MA2	1032	0.41	0.44	0.89	0.73	0.50	82
MA3	1234	0.50	0.41	0.94	0.72	0.60	83
CMA2	969	0.37	0.46	0.45	0.71	0.44	84
CMA4	1975	0.76	0.53	0.79	0.74	0.87	87
HMA100-2	1069	0.40	0.32	0.91	0.56	0.55	73
HMA150-2	1154	0.45	0.44	0.85	0.69	0.62	73
HMA200-2	1317	0.51	0.43	0.83	0.71	0.70	73

^a^ Micropores (%) = [W_0_(N_2_)/V_0.95_] × 100.

**Table 4:** Capacitances from GCDs in 2 and 3EC at 1 A·g^−1^. C_max_ at 1 mHz, equivalent series resistance (ESR), charge transfer resistance (R_CT_) and relaxation time constant (τ) from EIS in 2EC.

Sample	3EC	2EC					
	C (F·g^−1^)	C (F·g^−1^)	CA (μF·cm^−2^)	Cmax (F·g^−1^)	ESR (Ω)	R_CT_ (Ω)	τ (s)
MA1	149	140	16.9	117	0.41	0.74	1.98
MA2	244	232	22.5	236	0.41	0.71	1.48
MA3	246	240	19.4	235	0.32	0.67	1.11
CMA2	178	131	13.5	159	0.50	2.39	1.48
CMA4	257	240	12.2	261	0.40	2.11	1.11
HMA100-2	185	157	14.7	169	0.52	1.74	1.48
HMA150-2	260	207	17.9	227	0.44	2.08	1.48
HMA200-2	256	202	15.3	205	0.32	1.53	0.83

**Table 5:** Energy density (Wh·Kg^−1^) at 110 W·kg^−1^ from Ragone plots.

Sample	Energy Density
MA1	20.0
MA2	21.2
MA3	27.4
CMA2	16.7
CMA4	20.8
HMA100-2	16.3
HMA150-2	18.6
HMA200-2	26.1

## Supplementary Materials

The following are available online at www.mdpi.com/1996-1944/10/7/747/s1, Figure S1: ESEM images of precursors, Figure S2: N_2_ adsorption isotherms at −196 K, Figure S3: CVs at 2.5 mV·s^−1^ and GCDs at 1 A·g^−1^, Figures S4–S6: CVs at 0.5, 10, and 30 mV·s^−1^, Figure S7: Variation of gravimetric capacitance with current density, Figure S8: Evolution of imaginary part of capacitance vs. frequency.
