# Supplementary material for: Symmetric Supercapacitor Electrodes from KOH Activation of Pristine, Carbonized, and Hydrothermally Treated Melia azedarach Stones
Source: Materials (Basel). 2017 Jul 4;10(7):747. doi: 10.3390/ma10070747 (PMC5551790; doi:10.3390/ma10070747)
Supplement: Supplementary file 1 [file materials-10-00747-s001.pdf]

materials MDPI

Supplementary Materials

Symmetric Supercapacitor Electrodes from KOH Activation of Pristine, Carbonized and Hydrothermally Treated *Melia Azedarach* Stones

Carlos Moreno-Castilla*, Helena García-Rosero, and Francisco Carrasco-Marín

Departamento de Química Inorgánica, Universidad de Granada, 18071 Granada, Spain
* Corresponding author: E-mail: cmoreno@ugr.es

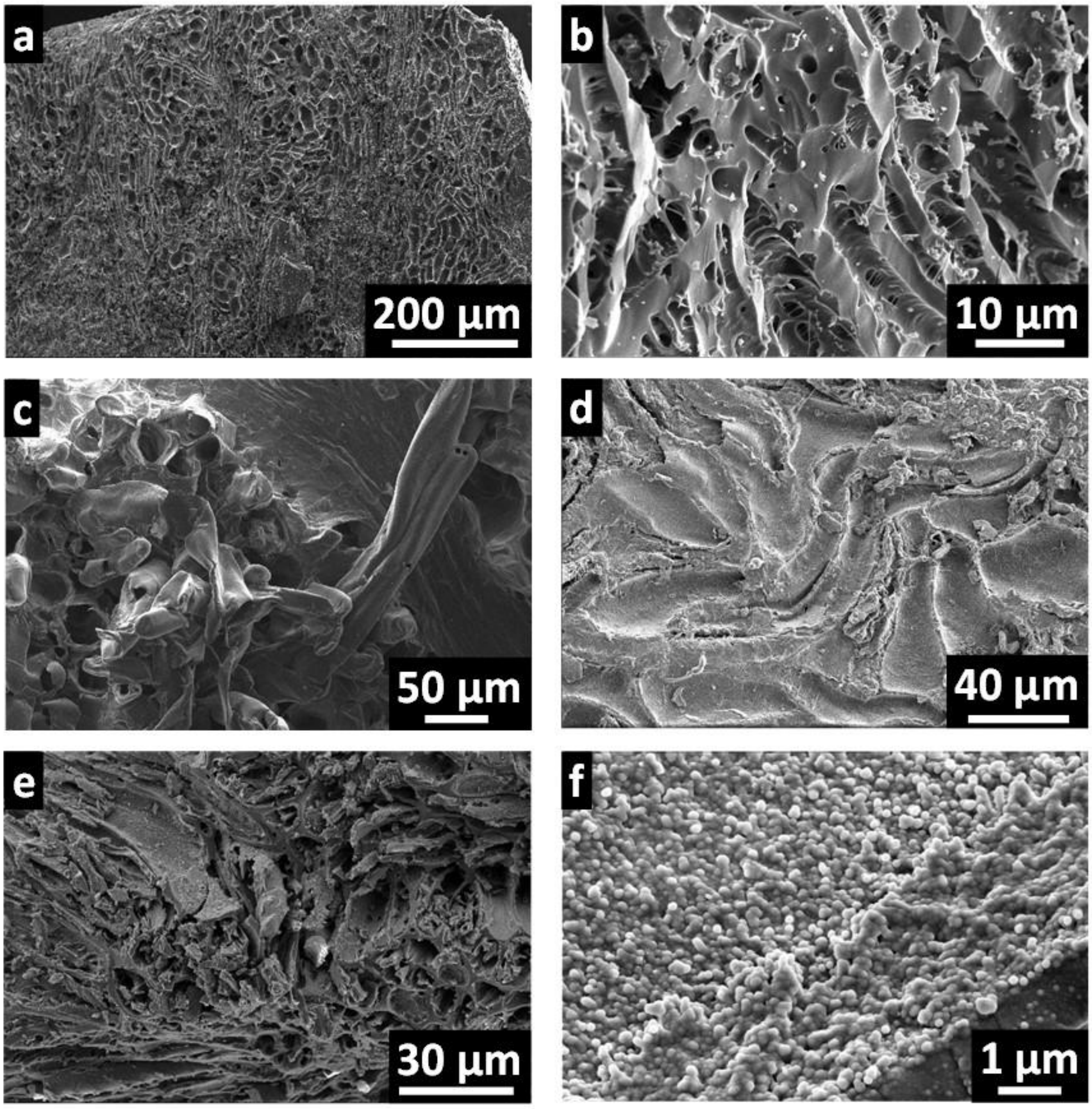

Figure S1. ESEM images of precursors: (**a**,**b**) CM; (**c**) HMA100; (**d**) HMA150; and (**d**,**f**) HMA200.

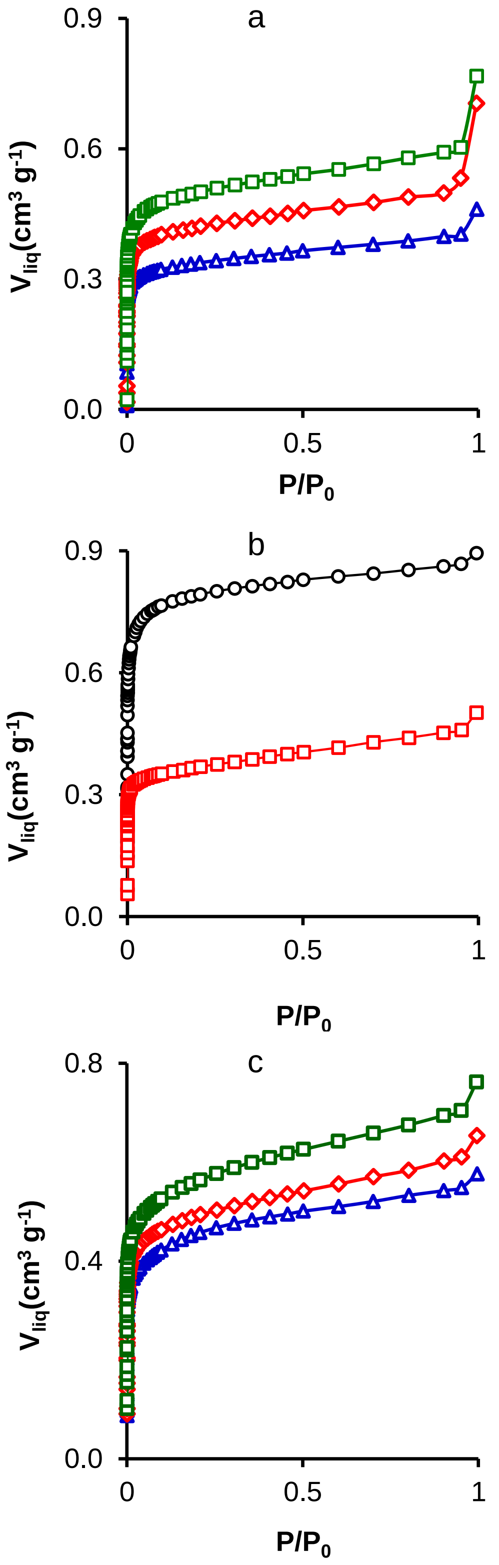

Figure S2. N_2 adsorption isotherms at −196 °C of samples: (**a**) MA1 (△), MA2 (◇), and MA3 (□); (**b**) CMA2 (□), and CMA4 (○); and (**c**) HMA100-2 (△), HMA150-2 (◇), and HMA200-2 (□).

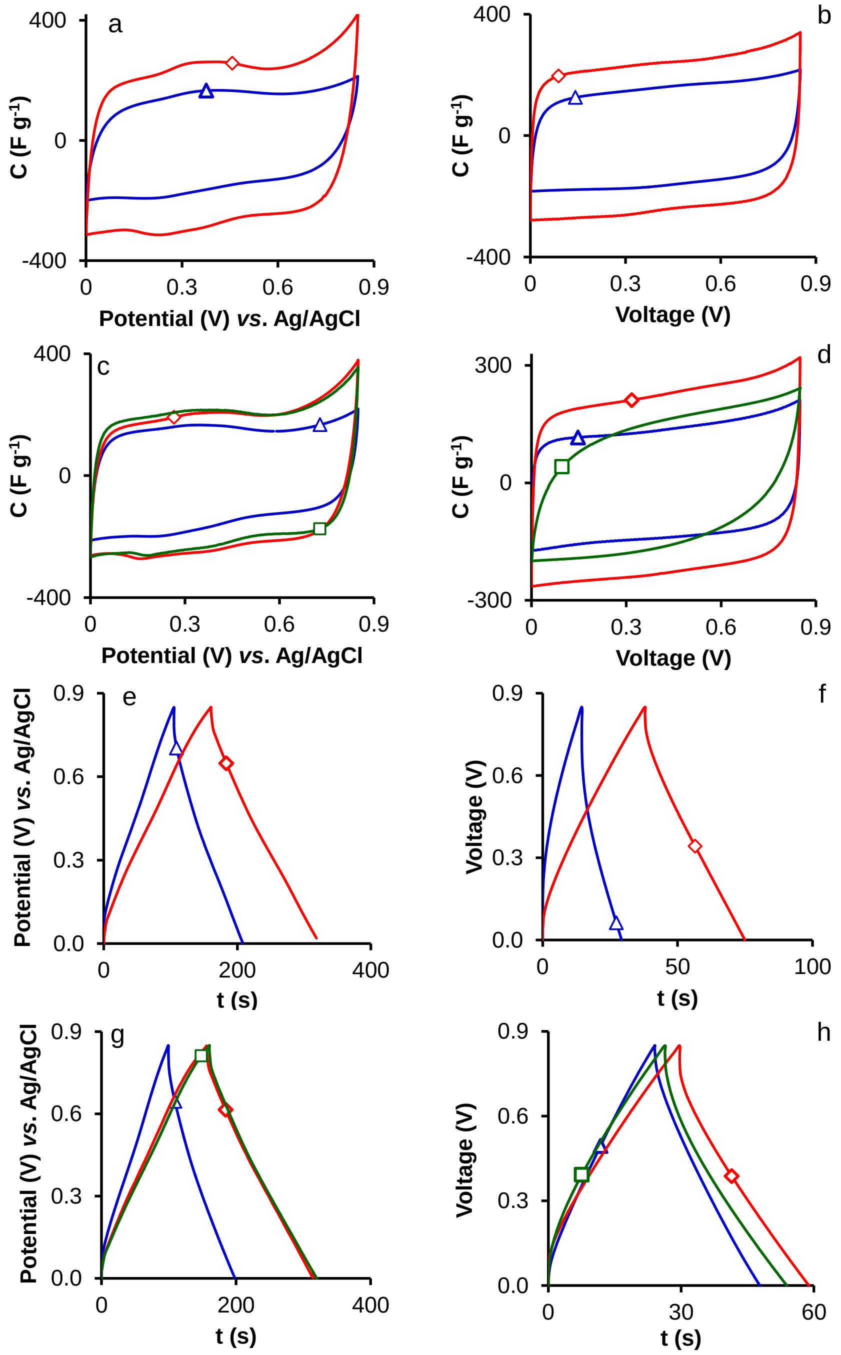

Figure S3. CVs at 2.5 mV·s^{-1} of samples (**a**,**b**) CMA2 (△), and CMA4 (◇); and (**c**,**d**) HMA100-2 (△), HMA150-2 (□), and HMA200-2 (◇). GCDs at 1 A·g^{-1} of samples (**e**,**f**) CMA2 (△), and CMA4 (◇); and (**g**,**h**) HMA100-2 (△), HMA150-2 (□), and HMA200-2 (◇).

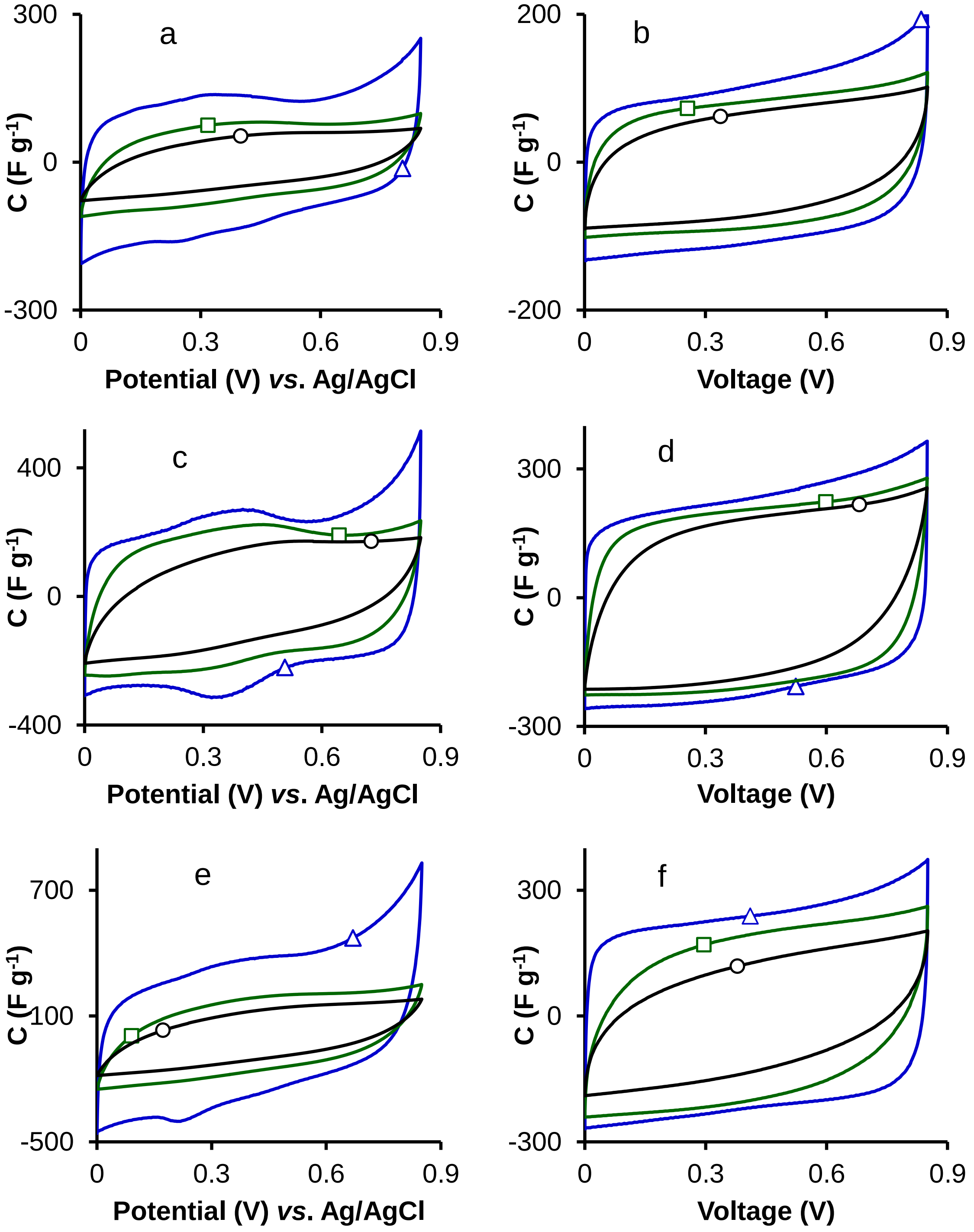

Figure S4. CVs at 0.5 (△), 10 (□), and 30 (○) mV·s^{-1} of samples: (**a**,**b**) MA1; (**c**,**d**) MA2; and (**e**,**f**) MA3.

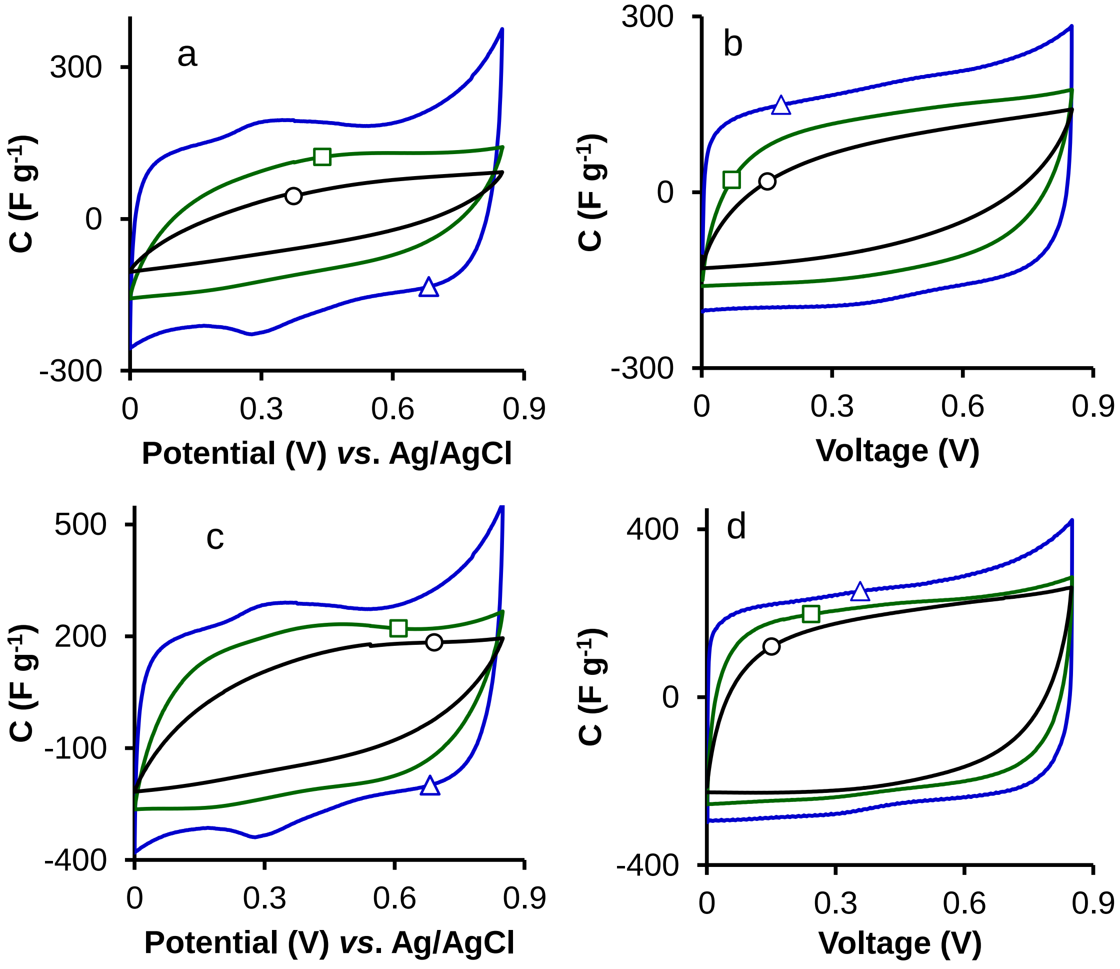

Figure S5. CVs at 0.5 (△), 10 (□), and 30 (○) mV·s^{-1} of samples: (**a**,**b**) CMA2; and (**c**,**d**) CMA4.

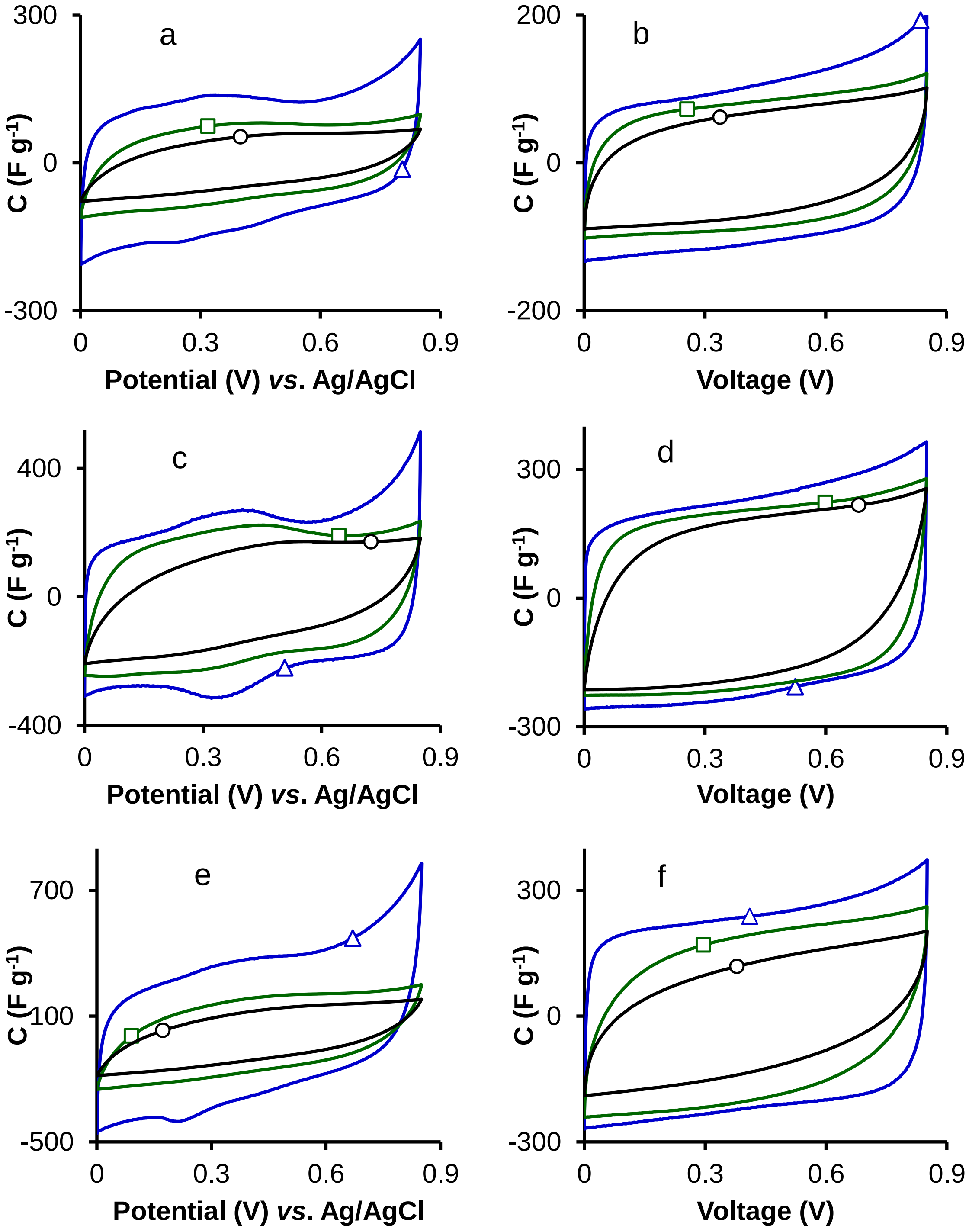

Figure S6. CVs at 0.5 (△), 10 (□), and 30 (○) mV·s^{-1} of samples: (**a**,**b**) HMA100-2; (**c**,**d**) HMA150-2; and (**e**,**f**) HMA200-2.

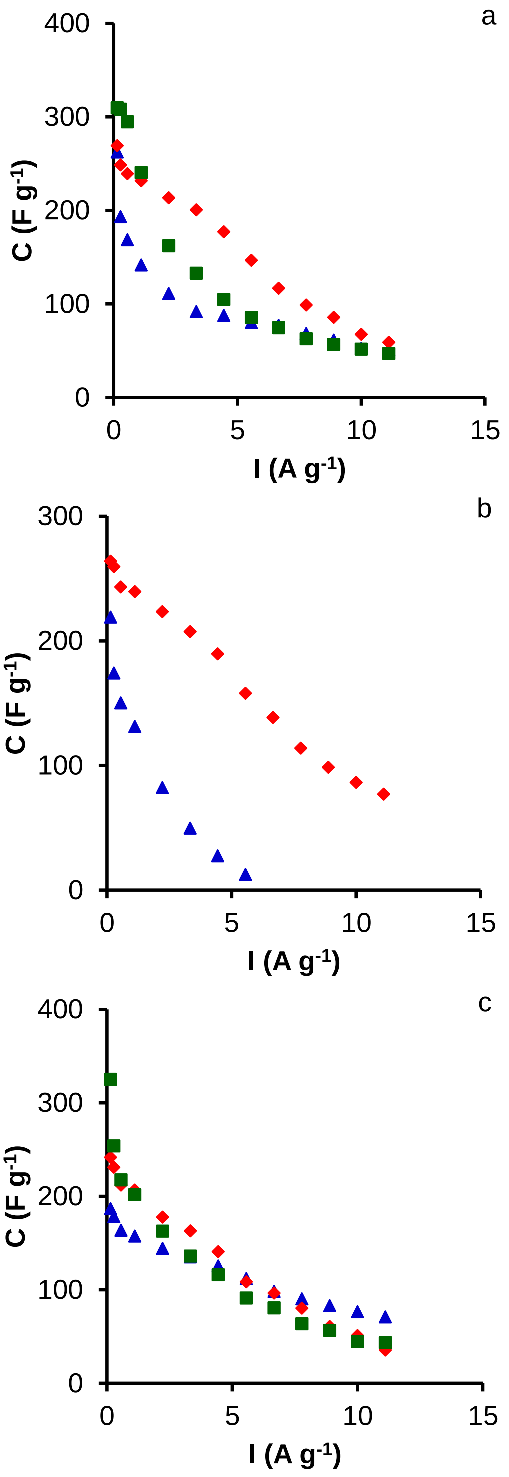

Figure S7. Variation of gravimetric capacitance with current density: (**a**) MA1 (▲), MA2 (◆), and MA3 (■); (**b**) CMA2 (▲), and CMA4 (◆); and (**c**) HMA100-2 (▲), HMA150-2 (◆), and HMA200-2 (■).

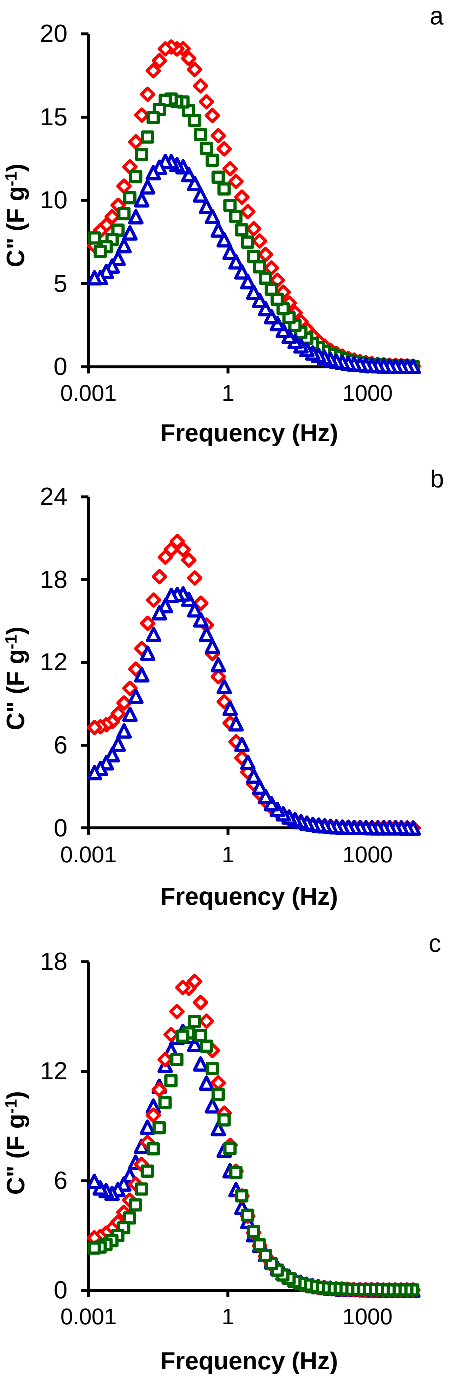

Figure S8. Evolution of imaginary part of capacitance *vs*. frequency. (**a**) MA1 (△), MA2 (□), and MA3 (◇); (**b**) CMA2 (△), and CMA4 (◇); and (**c**) HMA100-2 (△), HMA150-2 (□), and HMA200-2 (◇).
